# Association of low-density lipoprotein/high-density lipoprotein ratio with cognition, Alzheimer’s disease biomarkers and brain structure

**DOI:** 10.3389/fnagi.2025.1457160

**Published:** 2025-04-30

**Authors:** Zihao Zhang, Lingzhi Ma, Liangyu Huang, Yangke Zhu, Haihua Guo, Lanyang Wang, Wei Zhang, Lan Tan

**Affiliations:** ^1^Department of Neurology, Qingdao Municipal Hospital, Qingdao University, Qingdao, China; ^2^Department of Neurology, Qingdao Municipal Hospital, Nanjing Medical University, Nanjing, China

**Keywords:** Alzheimer’s disease, low-density lipoprotein/high-density lipoprotein, pathology, cognition, brain structure

## Abstract

**Background:**

The relationship between the low-density lipoprotein/high-density lipoprotein (LDL/HDL) ratio, an indicator of lipid metabolism and assessment of cardiovascular disease (CVD) risk, and Alzheimer’s disease (AD) is unclear.

**Methods:**

Multivariable Cox regression analyses were used to examine the association of LDL/HDL ratio and the risk of AD. Multiple linear regression and mixed effects models used to assess associations between LDL/HDL and cognitive function, AD pathology, and brain structure. Mediation analyses examined AD pathology’s potential mediating role between the LDL/HDL ratio and cognition.

**Results:**

Higher LDL/HDL ratio correlated with lower AD risk (HR 0.644 [0.431, 0.962]). In the linear regression analyses, the LDL/HDL ratio were positively associated with cognition. Longitudinally, the LDL/HDL ratio also positively with cognitive measures. Besides, higher LDL/HDL ratio were associated Aβ_42_ and decreased Tau, pTau, Tau/Aβ_42_, pTau/Aβ_42_, and pTau/Tau. The LDL/HDL ratio was positively associated with brain structures such as hippocampal volume. Mediation analyses revealed AD pathology mediated the association between LDL/HDL ratio and cognition.

**Conclusion:**

The LDL/HDL ratio is associated with AD risk, cognition, AD biomarkers and brain structure and can affect cognition by AD biomarkers.

## Introduction

Alzheimer’s disease (AD) is a neurodegenerative condition marked by gradual cognitive deterioration and hindered daily living capabilities, imposing a tremendous burden on individuals and society (Xu et al., 2015; Yang et al., 2013). Recent studies have shown that abnormalities in lipid metabolism are closely associated with the onset and progression of AD (de Bruijn and Ikram, 2014). Reduced levels of high-density lipoprotein (HDL) and increased levels of low-density lipoprotein (LDL) are frequently observed abnormalities in lipid metabolism, and they are closely associated with vascular dysfunction, inflammatory responses, and oxidative stress, which may contribute to the development of AD.

The National Cholesterol Education Program outlines target LDL and HDL cholesterol levels for assessing heart disease risk (Expert Panel on Detection, Evaluation, and Treatment of High Blood Cholesterol in Adults, 2001) The LDL/HDL ratio, a key indicator of lipid balance, reflects both atherogenic and cardioprotective lipid fractions, making it an effective metric for evaluating overall cardiovascular health and predicting cardiovascular events (Arsenault et al., 2011; Fernandez and Webb, 2008). Elevated LDL/HDL ratios have been strongly associated with coronary heart disease risk, atherosclerosis, and other cardiovascular conditions (Schnabel et al., 2010). This underscores the ratio’s utility in assessing not only heart disease risk but also potential implications for cerebral health.

Moreover, increasing evidence suggests that cardiovascular disease (CVD) is an important risk factor for AD (Saeed et al., 2023). Vascular pathology may accelerate the progression of AD by reducing cerebral perfusion, disrupting the integrity of the blood–brain barrier (BBB), and promoting the deposition of amyloid-β and tau proteins (Stakos et al., 2020). Therefore, evaluating lipid-related cardiovascular risk factors may play a significant role in the prediction and prevention of AD.

Given the robust predictive value of the LDL/HDL ratio for cardiovascular conditions and the established link between cardiovascular health and cognitive function, it is imperative to examine the potential association between the LDL/HDL ratio and Alzheimer’s disease. Despite known correlations, little research has specifically addressed how the LDL/HDL ratio relates to cognitive function, AD biomarkers, and brain structure. This study aims to fill this gap by exploring the relationships between the LDL/HDL ratio, AD risk, biomarkers, cognitive measures, and brain structure, and to elucidate the mechanisms by which lipid metabolism might influence cognitive functions, thereby providing insights into the interconnected pathways of lipid metabolism, cardiovascular health, and neurodegeneration.

## Methods

### Participants

A total of 616 non-dementia adults were gathered from the Alzheimer’s Disease Neuroimaging Initiative (ADNI). These participants, ranging in age from 55 to 90 years, provided us with comprehensive data from the ADNI study, including essential clinical characteristics, biochemical biomarkers of AD, imaging data, and cognitive assessment data. Participants with missing LDL, HDL, ethnicity, marriage, education, *APOEε4* carrier status, cognitive diagnosis, systolic, diastolic, total cholesterol (TC), diabetes and CVD data and dementia were excluded. More information is available at http://adni.loni.usc.edu (Petersen et al., 2010).

In addition, we examined data from the CABLE study from 1,105 adults without dementia. The CABLE experiment is detailed in the Supplementary file. Patients with missing LDL, HDL, cerebrospinal fluid (CSF) biomarkers, cognitive measures, and covariate data were excluded from the study, in addition to patients with dementia. In the CABLE database, in addition to the previous exclusion criteria, those with >15% variability in CSF batch were also excluded from the study.

### Laboratory parameters

The ADNI database provided all laboratory and anthropometric parameters and medical history data. LDL and HDL levels were measured. *APOEε4* genotyping was conducted at the ADNI Biomarker Core Laboratory, University of Pennsylvania, to identify participants with at least one *APOEε4* allele, designating them the *APOEε4* positive status (Shaw et al., 2009). Since the majority of the included population lacked a diagnosis of diabetes, we used fasting blood glucose (FDG) levels instead of missing components, and an FDG ≥ 7.0 mmol/L was considered to have diabetes (American Diabetes Association, 2021).

### CSF AD biomarkers

Cerebrospinal fluid samples were collected and placed in 10-mL polypropylene tubes using a lumbar puncture. Within 2 h, these samples were transported to the laboratory. Subsequently, the CSF samples underwent centrifugation at 2,000 *g* for 10 min. Samples not detected in time for thawing and freezing procedures did not exceed two cycles prior to analysis (Shaw et al., 2009; Sheng et al., 2025). The INNO-BIA AlzBio3 immunoassay (Innogenetics-Fujirebio, Ghent, Belgium) was used to measure CSF Aβ_42_, Tau, and phosphorylated Tau (pTau181) concentrations (pg/mL).

The procedures for collecting CSF have previously been documented in CABLE. To quantify CSF levels of Aβ_40_, Aβ_42_, Tau, and pTau, an enzyme-linked immunosorbent assay kit (Innotest; Fujirebio, Ghent, Belgium) was used on a microplate reader (Multiskan MK3; Thermo Fisher Scientific, Waltham, MA) (Zhang et al., 2024).

Incorporating Tau/Aβ_42_ and pTau/Aβ_42_ ratios is crucial, given their enhanced predictive value for brain Aβ deposition and cognitive decline compared to their individual expression (Racine et al., 2016; Snider et al., 2009).

### Definition of incident AD and cognitive measures

Alzheimer’s disease was diagnosed in patients who met the criteria for probable AD established by the National Institute of Neurological and Communicative Disorders and Stroke and the Alzheimer’s Disease and Related Disorders Association (McKhann et al., 1984). The ADNI used a series of scales to assess cognitive functions, encompassing assessments of global cognition using the Mini-Mental State Examination (MMSE) and the cognitive domain of the Alzheimer’s Disease Assessment Scale (ADAS), in addition to specific cognitive domains such as episodic memory (MEM), executive function (EF), language (LAN), and visual–spatial functions (VS) (Fletcher et al., 2021). Higher ADAS scores and lower MMSE, MEM, EF, LAN, and VS scores indicate poorer cognitive functioning.

### Neuroimaging

Brain structural magnetic resonance imaging (MRI) data were acquired using either a Siemens Trio 3.0-T or a Vision 1.5-T imaging system. The image analysis software Freesurfer (versions 4.3 and 5.1) was utilized for processing to estimate regional brain volumes from the MRI images (Jack et al., 2008; Zhang et al., 2024). Our analyses included measurements of whole brain volume hippocampus, middle temporal, and entorhinal cortex.

### Statistical analyses

For continuous variables, mean (standard deviation) or median (interquartile range) were used to represent normal or non-normal distributions, respectively. Analysis of Variance or the Kruskal–Wallis test was employed for analysis accordingly. Categorical variables were depicted as numbers (n) and percentages (%), with chi-square tests employed for assessment. Outliers exceeding three standard deviations were eliminated from the statistical analysis involving the LDL/HDL ratio. Relationships between the LDL/HDL ratio and AD biomarkers, cognition, and brain structure were explored via multiple linear regression. Subgroup analyses were conducted based on age, sex, ethnicity, *APOEε4* carrier status, cognitive diagnosis, diabetes, CVD and hypercholesterolemia. Multicollinearity was assessed, and all variance inflation factors (VIFs) were below 2, indicating no significant collinearity among covariates (Supplementary Table 1). In addition, we used data from the CABLE cohort (currently limited to cross-sectional data, with longitudinal follow-up ongoing) to confirm the associations between the LDL/HDL ratio and AD biomarkers and cognitive function observed in the ADNI dataset.

To explore the relationship between the LDL/HDL ratio and the risk of AD, we used the Cox proportional risk model to estimate the hazard ratio (HR) and 95% confidence interval (CI) for AD. Restricted cubic spline (RCS) regression analyses were employed to examine the relationships between LDL/HDL ratio and AD risk. Linear mixed-effects models were then employed to delineate the longitudinal relationships among the LDL/HDL ratio, AD pathology, cognition, and brain structure.

Mediation analyses, following Baron and Kenny’s approach, were conducted to ascertain whether AD pathology mediated the association of the LDL/HDL ratio with cognition (Baron and Kenny, 1986). In addition, the magnitude of attenuation or indirect effects was estimated and significance was ascertained via 10,000 self-directed iterations.

In all analyses, adjustments were made for age, sex, ethnicity, marriage, education, *APOEε4* carrier status, cognitive diagnosis, systolic, diastolic, total cholesterol, diabetes and cardiovascular disease as covariates. Given that all outcome variables were standardized to *z*-scores in the model, the coefficient represents the standardized effect. Statistical analyses were performed using R version 4.2.0, with statistical significance set at *p* < 0.05 for all analyses.

## Results

### Participants’ characteristics

In the ADNI database, 616 participants were included, with a mean age of 73.20 ± 7.10 years. Among them, 43.3% were female, and 66.9% had a history of CVD. Our analysis revealed that the mean LDL/HDL ratio was 1.27 ± 0.35 in the group with normal cognition, which was lower compared to those with MCI (1.36 ± 0.38) (Table 1).

**Table 1:** Characteristics based on the LDL/HDL ratio of 616 participants obtained from the Alzheimer’s Disease Neuroimaging Initiative (ADNI).

Variance	Overall	CN	MCI	*P*
Number	616	171	445	
Age (years)	73.20 (7.10)	74.70 (6.28)	72.62 (7.32)	0.001
Education (years)	16.19 (2.74)	16.23 (2.73)	16.18 (2.75)	0.838
Sex (%)				0.089
Female	267 (43.3)	84 (49.1)	183 (41.1)	
Male	349 (56.7)	87 (50.9)	262 (58.9)	
Ethnicity (%)				0.064
Other	38 (6.2)	16 (9.4)	22 (4.9)	
White	578 (93.8)	155 (90.6)	423 (95.1)	
Marriage (%)	51 (8.3)	12 (7.0)	39 (8.8)	0.011
Divorced	51 (8.3)	12 (7.0)	39 (8.8)	
Married	478 (77.6)	122 (71.3)	356 (80.0)	
Never married	18 (2.9)	8 (4.7)	10 (2.2)	
Widowed	69 (11.2)	29 (17.0)	40 (9.0)	
APOE*ε*4 (%)				<0.001
Non-carry	319 (51.8)	119 (69.6)	200 (44.9)	
Carry	297 (48.2)	52 (30.4)	245 (55.1)	
Smoking				0.864
No	369 (59.9)	101 (59.1)	268 (60.2)	
Yes	247 (40.1)	70 (40.9)	177 (39.8)	
Cardiovascular (%)				0.459
No	204(33.1)	61(35.7)	143(32.1)	
Yes	412(66.9)	110(64.3)	302(67.9)	
Diabetes (%)				0.074
No	532 (86.4)	155 (90.6)	377 (84.7)	
Yes	84 (13.6)	16 (9.4)	68 (15.3)	
Hypercholesterolemia				0.967
No	538 (87.3)	150 (87.7)	388 (87.2)	
Yes	78 (12.7)	21 (12.3)	57 (12.8)	
Systolic pressure (mmHg)	135.63 (16.38)	134.59 (15.73)	136.03 (16.62)	0.330
Diastolic pressure (mmHg)	75.37 (9.64)	75.64 (9.80)	75.27 (9.59)	0.674
TC (mmol/L)	4.94 (0.94)	4.90 (0.94)	4.96 (0.95)	0.508
LDL (mmol/L)	1.95 (0.44)	1.90 (0.43)	1.97 (0.45)	0.086
HDL (mmol/L)	1.53 (0.38)	1.57 (0.40)	1.51 (0.37)	0.075
LDL/HDL	1.34 (0.37)	1.27 (0.35)	1.36 (0.38)	0.004
Aβ_42_ (pg/ml)	898.37 (367.96)	1029.61 (379.38)	847.94 (351.07)	<0.001
Tau (pg/ml)	272.42 (127.31)	226.13 (88.46)	290.20 (135.33)	<0.001
pTau (pg/ml)	26.50 (14.25)	21.18 (9.51)	28.55 (15.22)	<0.001
pTau/Aβ_42_	0.04 (0.03)	0.03 (0.02)	0.04 (0.03)	<0.001
Tau/Aβ_42_	0.38 (0.27)	0.27 (0.18)	0.42 (0.28)	<0.001
pTau/Tau	0.09 (0.01)	0.09 (0.01)	0.10 (0.01)	<0.001
MMSE score	28.10 (1.78)	29.13 (1.08)	27.70 (1.83)	<0.001
ADAS score	9.08 (4.78)	5.99 (3.06)	10.27 (4.79)	<0.001
EF score	0.34 (0.90)	0.74 (0.79)	0.19 (0.90)	<0.001
MEM score	0.41 (0.78)	1.05 (0.57)	0.17 (0.70)	<0.001
LAN score	0.38 (0.80)	0.81 (0.71)	0.22 (0.77)	<0.001
VS score	0.02 (0.70)	0.21 (0.59)	−0.06 (0.72)	<0.001
Hippocampus (mm^3^)	6961.79 (1112.98)	7428.61 (829.71)	6780.73 (1156.26)	<0.001
Entorhinal (mm^3^)	3610.77 (724.90)	3827.35 (608.71)	3524.71 (749.72)	<0.001
Middle temporal (mm^3^)	20058.75 (2852.70)	20511.94 (2585.41)	19878.66 (2936.04)	0.021

Data are mean (SD), *n* (%), or median (IQR).

LDL/HDL, low-density lipoprotein/high-density lipoprotein; APOEε4, apolipoprotein E4; CN, cognition normal; MCI, mild cognition mild; TC, total cholesterol; Aβ_42_, amyloid-42; pTau, phosphorylated-tau; Tau, total-tau; MMSE, mini-mental state examination, ADAS, Alzheimer’s Disease Assessment Scale; EF, executive function; MEM, memory function; LAN, language; VS, visuospatial functioning.

In the CABLE database, 1,105 individuals were included, with a mean age of 62.96 ± 10.06 years. Among them, 42.7% were female, and 44.3% had a history of CVD (Supplementary Table 2).

### LDL/HDL ratio and AD risk

During a follow-up period of up to 16 years, we analyzed patient groups with different LDL/HDL ratio using Cox proportional risk model. In the adjusted risk model, we observed that for each unit increase in the LDL/HDL ratio, the risk of developing AD decreased by 35.6% (HR 0.644, 95% CI [0.431, 0.962]). Furthermore, in women, individuals who did not carry the *APOEε4* gene, MCI participate and diabetes, we observed a decreased risk of AD by 58.0% (HR 0.420, 95% CI [0.200, 0.866]), 61.7% (HR 0.383, 95% CI [0.191, 0.765]), 34.9% (HR 0.651, 95% CI [0.426, 0.993]) and 72.5% (HR 0.275, 95% CI [0.078, 0.963]) respectively, with increasing LDL/HDL ratio (Table 2). RCS results did not find a non-linear relationship between LDL/HDL ratio and risk of AD (Supplementary Figure 1).

**Table 2:** Risk factors associated with Alzheimer’s Disease development using Cox regression.

Variance		HR (95%CI)	*P*
Total		0.644 (0.431, 0.962)	**0.032**
Age	<60		
	≥60	0.670 (0.446, 1.008)	0.055
Sex	Male	0.751 (0.456, 1.236)	0.260
	Female	0.420 (0.200, 0,866)	**0.019**
APOE*ε*4	APOE*ε*4 (−)	0.383 (0.191, 0.765)	**0.007**
	APOE*ε*4 (+)	0.866 (0.521, 1.437)	0.577
Diagnosis	CN	0.858 (0.160, 4.604)	0.858
	MCI	0.651 (0.426, 0.993)	**0.047**
Cardiovascular	No	0.545 (0.279, 1.066)	0.076
	Yes	0.655 (0.389, 1.103)	0.112
Diabetes	No	0.681 (0.438, 1.058)	0.088
	Yes	0.275 (0.078, 0.963)	**0.044**
Hypercholesterolemia	No	0.641 (0.403, 1.018)	0.059
	Yes	0.753 (0.210, 2.700)	0.663

All factors adjusted for age, sex, ethnicity, marriage, education, APOEε4 carrier status, smoking, cognitive diagnosis, systolic, diastolic, total cholesterol, diabetes and cardiovascular disease.

Not included in the analyses due to the small number of persons younger than 60 years of age. Bold values denote statistical significance at *p* < 0.05.

### Association of LDL/HDL ratio with cognitive measures

**Figure 1:**
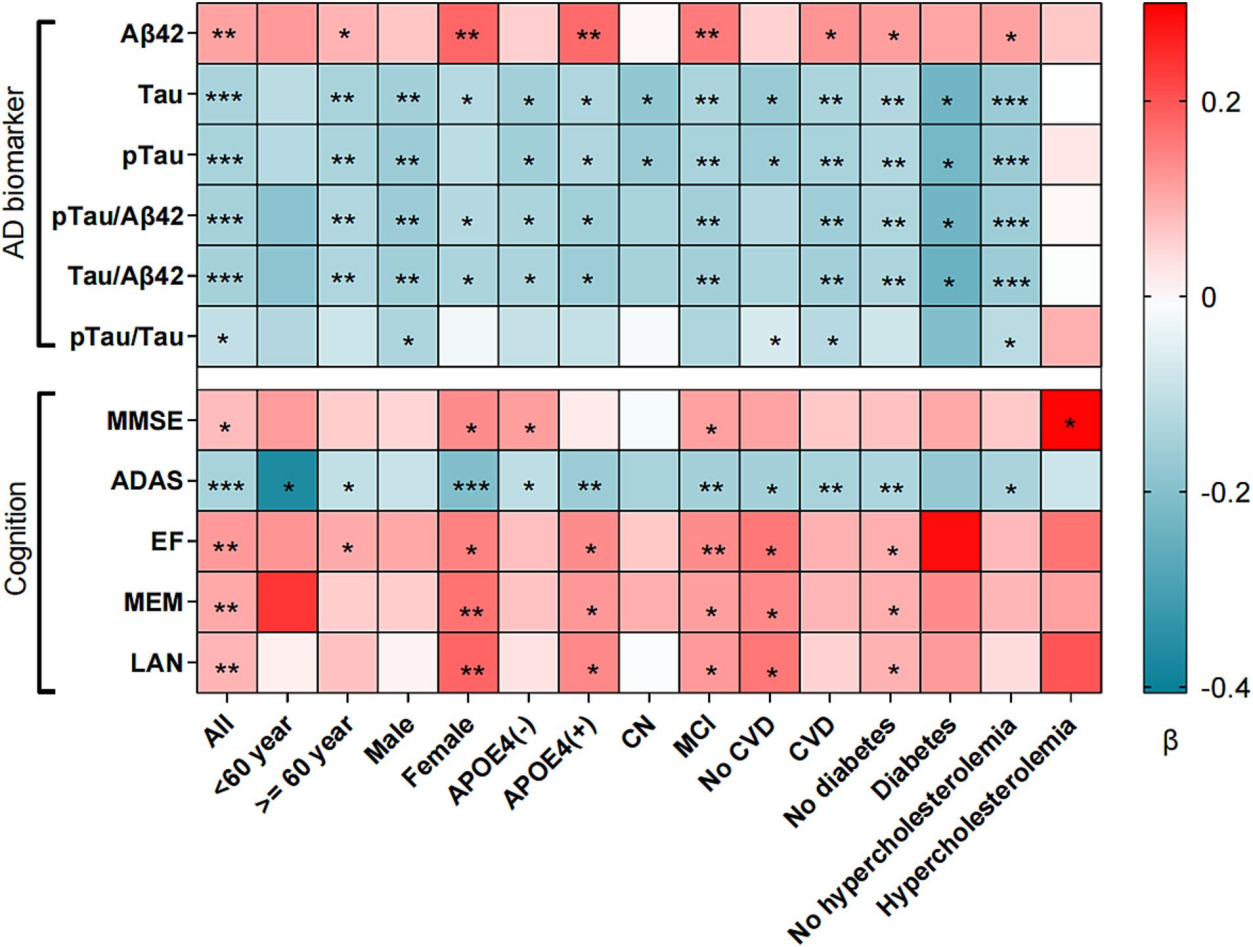
Association between LDL/HDL ratio and cognition, AD biomarker and brain structure. LDL/HDL, low-density lipoprotein/high-density lipoprotein; AD, Alzheimer’s disease; Aβ_42_, Amyloid-42; pTau, phosphorylated-tau; Tau, total-tau; MMSE, mini-mental state examination, ADAS, Alzheimer’s Disease Assessment Scale; EF, executive function; MEM, memory function; LAN, language; VS, visuospatial functioning; APOE*ε*4, apolipoprotein E4; CN, cognition normal; MCI, mild cognition mild; CVD, cardiovascular disease. *p < 0.05, **p < 0.01, ***p < 0.001. All factors adjusted for age, sex, ethnicity, marriage, education, APOE*ε*4 carrier status, smoking, cognitive diagnosis, systolic, diastolic, total cholesterol, diabetes and cardiovascular disease.

In the ADNI database, the LDL/HDL ratio showed positive correlations with MMSE (*β* = 0.080, *p* = 0.048), EF (*β* = 0.118, *p* = 0.004), MEM (*β* = 0.101, *p* = 0.006), and LAN (*β* = 0.086, *p* = 0.034), while exhibiting negative correlations with ADAS (*β* = −0.120, *p* = 0.002) (Figure 1 and Supplementary Table 3). Subgroup analyses revealed significant associations between LDL/HDL ratio and cognitive function, in older adults aged over 60, females, participants carrying the *APOEε4* gene, individuals with MCI, and participants without diabetes. In additional, we investigated longitudinal associations between LDL/HDL ratio and cognitive function, we found that the LDL/HDL ratio was positively associated with MMSE scores (*β* = 0.026, *p* = 0.047), MEM scores (*β* = 0.015, *p* = 0.033), LAN scores (*β* = 0.017, *p* = 0.048) and negatively associated with ADAS scores (*β* = −0.020, *p* = 0.050). These findings suggest potential links between LDL/HDL ratio and changes in cognitive function overtime (Supplementary Table 4).

### Association of LDL/HDL ratio with CSF AD biomarkers

**Figure 2:**
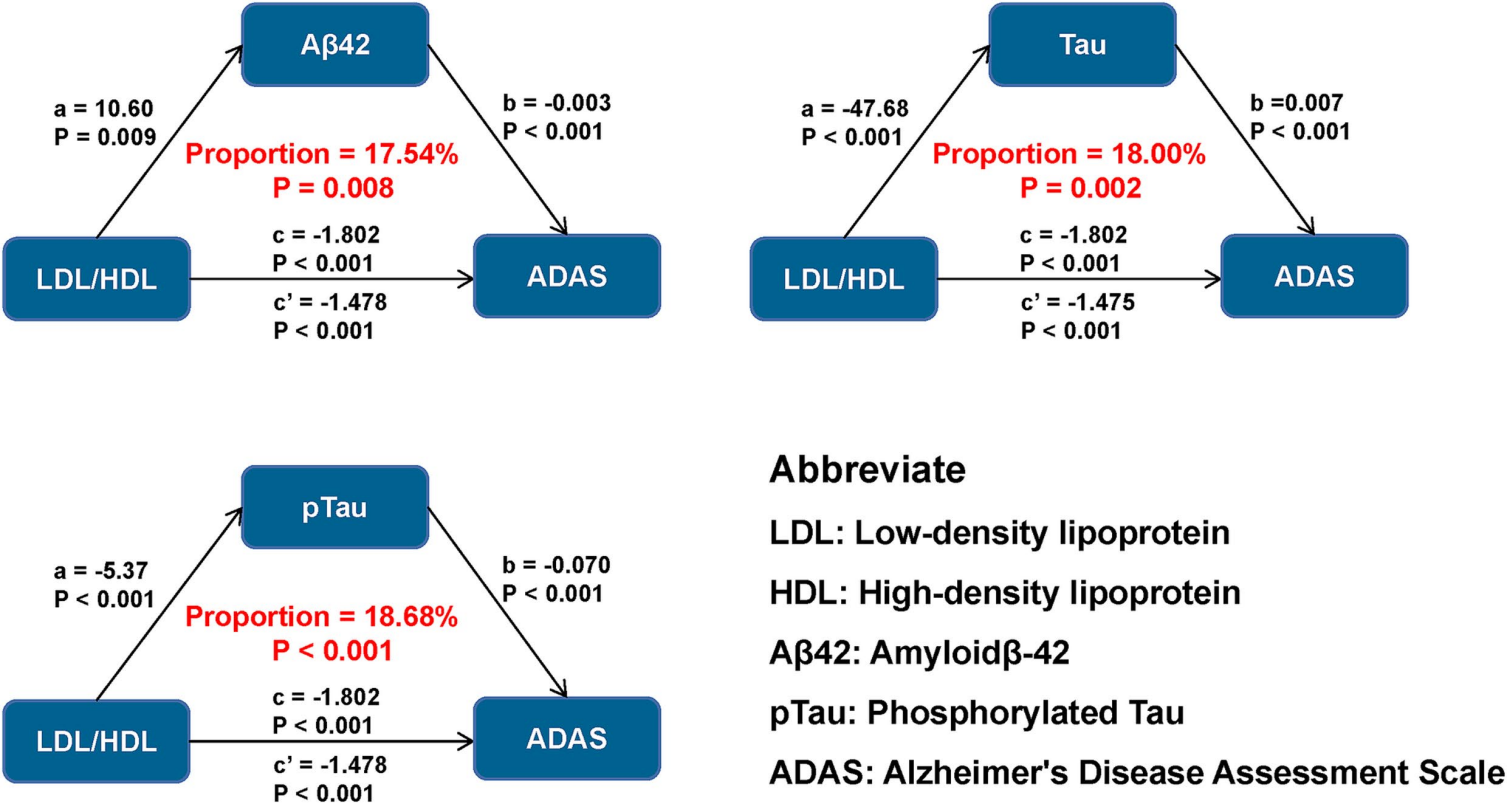
Mediation analyses of LDL/HDL ratio and baseline cognition with AD biomarkers as mediators. LDL/HDL, low-density lipoprotein/high-density lipoprotein; AD, Alzheimer’s disease; Aβ_42_, amyloid-42; pTau, phosphorylated-tau; Tau, total-tau; MMSE, mini-mental state examination, ADAS, Alzheimer’s Disease Assessment Scale; EF, executive function; MEM, memory function; LAN, language; VS, visuospatial functioning.

In the ADNI database, higher LDL/HDL ratios are associated with higher levels of Aβ_42_ (*β* = 0.108, *p* = 0.009). Conversely, with increasing LDL/HDL ratio, levels of Tau (*β* = −0.140, *p* < 0.001), pTau (*β* = −0.141, *p* < 0.001), Tau/Aβ_42_ (*β* = −0.147, *p* < 0.001), pTau/Aβ42 (*β* = −0.145, *p* < 0.001), and pTau/Tau (*β* = −0.101, *p* = 0.018) gradually decreased (Figure 2 and Supplementary Table 5).

In the CABLE database, the LDL/HDL ratio was also negatively correlated with the levels of Tau (*β* = −0.064, *p* = 0.046) protein and pTau (*β* = −0.085, *p* = 0.008) protein (Supplementary Table 6). Similarly, in the CABLE database, we observed a negative association between the LDL/HDL ratio and the levels of Tau protein and pTau protein in older adults aged over 60, women, participants carrying the *APOEε4* gene, participants without diabetes, and participants without hypercholesterolemia, which is consistent with the findings from the ADNI (Supplementary Table 6).

### Association of LDL/HDL ratio with brain structure

Analysis of brain images showed that LDL/HDL was positively correlated with hippocampus (*β* = 0.102, *p* = 0.027), entorhinal cortex (*β* = 0.093, *p* = 0.041), and middle temporal volume (*β* = 0.104, *p* = 0.021). Subgroup analyses showed that LDL/HDL was also positively correlated with whole brain volume in those with MCI and diabetes mellitus (Supplementary Table 7).

### Causal mediation analyses

We examined whether the association between LDL/HDL ratio and cognitive measures was mediated by AD pathology. Our results suggest that the LDL/HDL ratio may exert direct effects on cognitive measures, as well as influence global cognition and specific cognitive domains by modulating Aβ_42_ levels, explaining between 17.54 and 27.19% of the total effect (Figure 2). Furthermore, the LDL/HDL ratio was associated with influence in cognitive function by decreasing Tau and pTau levels, as well as the ratios of pTau/Aβ_42_, Tau/Aβ_42_, and pTau/Tau, which accounted for 18.00–35.50%, 18.68–36.45%, 25.40–43.92%, 26.80–46.09%, and 11.54–19.05% of the total effect, respectively. Notably, the impact of LDL/HDL ratio on ADAS11 through AD pathology was relatively small compared to other cognitive measures analyzed, but had the greatest effect on MEM (Supplementary Table 8).

## Discussion

This study used found that LDL/HDL ratio was positively associated with CSF Aβ_42_ levels and inversely associated with Tau and pTau levels, and found the same trend in another database. Besides, LDL/HDL ratio was associated with cognitive performance. As for brain imaging, the LDL/HDL ratio was positively correlated with hippocampal, entorhinal cortex and middle temporal volume. There was a positive association between the LDL/HDL ratio and cognitive level over time, and the LDL/HDL ratio could also influence changes in cognitive level by affecting the three AD pathologic biomarkers and their ratios.

Research indicates that the relationship between CVD risk and cognitive function may differ across racial groups, potentially due to physiological, genetic, lifestyle, and environmental factors (Bailey et al., 2021). Notably, while the ADNI cohort predominantly consists of White participants and the CABLE cohort primarily includes Asians, our findings show similar trends in the association between LDL/HDL ratio and AD biomarkers (such as Tau and pTau) across both cohorts. This suggests that despite the ethnic differences, the underlying mechanisms linking lipid levels to AD pathology may be consistent.

Previous studies have been more inclined to consider the relationship between lipids and risk of AD development, cognitive measures, and brain imaging, and have paid less attention to direct associations between lipids and CSF biomarkers (Turri et al., 1867; Yu et al., 2023; Andrews et al., 2021). Our study bridges this gap. A study of a cognitively normal population reported no association between lipids and AD biomarkers (Jansson et al., 2022), which is inconsistent with our findings, and in addition to differences in the populations selected, the small sample size of this study may have been important in failing to find an association between lipids and AD (Peters et al., 2021).

Some studies suggest that higher LDL levels are associated with better memory function in older populations. For example, a study from Japan found that elevated LDL levels were linked to improved memory performance in elderly individuals (Katsumata et al., 2013). Similarly, a cohort study from China reported an inverse association between high LDL levels and dementia risk (Zhou et al., 2018). These findings align with the results of our study, which demonstrated a positive correlation between the LDL/HDL ratio and cognitive function. We hypothesize that the selective permeability of the BBB to different types of cholesterol may play a crucial role in this relationship (Poliakova and Wellington, 2023). HDL can cross the BBB through specific transport mechanisms, such as the SR-BI receptor, where it reduces Aβ deposition and suppresses inflammation, exerting neuroprotective effects. In contrast, LDL has limited ability to cross the BBB under normal physiological conditions, restricting its direct impact on the central nervous system (Rhea and Banks, 2021). This mechanism may explain why elevated peripheral LDL levels do not necessarily result in cognitive decline and may even be associated with better cognitive performance in certain elderly populations. Additionally, some studies suggest that higher LDL levels in older adults may reflect better nutritional status or overall health, whereas lower cholesterol levels could be linked to brain atrophy and poorer outcomes (Zhou et al., 2018; Pfrieger, 2003). Age-related differences may also contribute to these findings. A meta-analysis indicated that while high LDL levels may be a potential risk factor for AD, this association diminishes or disappears in populations over 70 years of age (Zhou et al., 2020). Similar to cognition, HDL is a protective factor for brain structure. HDL is positively correlated with volumes of the bilateral temporal pole, middle temporal gyrus and para-hippocampal regions (Ward et al., 2010); lower levels of HDL are associated with smaller hippocampal volumes (Wolf et al., 2004) LDL, on the other hand, is significantly correlated with decreased volume of total white matter and a decrease in the hippocampus (Wang et al., 2021).

Our mediation analysis suggests that the LDL/HDL ratio indirectly impacts cognitive function through its effects on CSF biomarkers such as Aβ_42_, Tau, and pTau. This modulation affects global cognition and specific cognitive domains, including executive function, memory, and language. Specifically, higher LDL/HDL ratios were associated with better performance in these cognitive domains by reducing Tau and pTau levels and increasing Aβ_42_ levels. Compared to other studies, our findings are consistent with those indicating that lipid levels influence AD progression through biochemical pathways involving these biomarkers. Previous research shows that elevated LDL increases AD risk, especially with *APOEε4*, while higher HDL levels are protective. These pathways likely involve the BBB and inflammatory processes, impacting biomarker levels and cognitive functions (Liu et al., 2021).

The LDL/HDL ratio, as an indicator for assessing CVD risk, an elevated ratio also reduces the risk of AD. It is worth noting that most participants in the ADNI database used in this study had LDL/HDL ratios below 2.5, which corresponds to a population with low CVD risk. This could explain the inverse association observed between LDL/HDL and AD risk in this study. Furthermore, a large proportion of participants were diagnosed with mild cognitive impairment (MCI). Early interventions, particularly the use of statins and dietary improvements, may have contributed to reducing AD risk. Statins not only lower peripheral cholesterol levels but also reduce neuroinflammation by inhibiting microglial activation and pro-inflammatory cytokine release. Additionally, they enhance BBB tegrity by stabilizing endothelial function, which may limit the entry of peripheral inflammatory mediators into the brain. These effects can reduce Aβ accumulation and tau hyperphosphorylation, thereby mitigating neurodegeneration (Zingel et al., 2021). Dietary improvements, such as adherence to heart-healthy patterns like the Mediterranean diet, have been associated with improved lipid profiles and reduced CVD risk (Middleton and Riboli, 2023).

Experimental and clinical evidence suggests that Aβ may play a role in the link between CVD and AD (Stakos et al., 2020), and Aβ_40_ levels were significantly and independently associated with subclinical atherosclerosis and coronary artery disease (Stamatelopoulos et al., 2015), suggesting a link between CVD and AD pathology. The higher the risk and severity of CVD, the more pronounced the impact on cognition. Studies have shown that there is an association between total CVD burden and cognitive deterioration (de Galan et al., 2009); the degree of cognitive impairment in patients with heart failure correlates with the severity of left ventricular systolic dysfunction (Zuccalà et al., 1997); and common CVDs such as stroke, hypertension, and coronary heart disease are associated with cognitive impairment (Abete et al., 2014). Our results show slow cognitive decline in people at low CVD risk. Song et al. (2020) found that a higher Framingham General Cardiovascular Risk Score was associated with smaller hippocampal volume and whole-brain volume, which is similar to our findings of low CVD risk with greater hippocampal volume.

The LDL/HDL ratio can influence the onset and progression of AD for the following reasons. Firstly, LDL and HDL can affect the BBB through a number of pathways. High levels of LDL may promote the formation of atherosclerosis, leading to damage to the arterial endothelium and the development of inflammatory responses (Pentikäinen et al., 2000). The inflammatory response leads to an increase in the permeability of vascular endothelial cells, which in turn affects the BBB (Galea, 2021). Second, an increase in the LDL/HDL ratio may directly affect the permeability of the BBB by influencing the composition and structure of the cell membrane (Muralidharan et al., 2022). LDL and HDL can affect the fluidity and stability of cell membranes, which in turn affects the permeability of the BBB (Rhea and Banks, 2021).

Our study has some strengths. First, we validated the association between the LDL/HDL ratio and biomarkers of AD in human CSF using two databases. Second, we illustrated the association between LDL/HDL ratio and AD from three aspects: pathology, cognitive measures, and brain imaging. Finally, the LDL/HDL ratio can affect global cognition and cognitive domains through three pathologic markers. Our study also has some limitations. First, our finding needs to be tested in a larger longitudinal cohort. Second, although we validated our findings in the CABLE cohort, differences in age distribution and cardiovascular characteristics between CABLE and ADNI mean that the results can only partially support the conclusions drawn from the ADNI cohort. In addition, although the LDL/HDL ratio could eliminate some of the effects of statins on LDL levels, some factors that can affect LDL, HDL levels such as the use of cholesterol-lowering and glucose-lowering medications, hormones, and diet, were not taken into account. Finally, the generalization of the findings is limited by the fact that participants in the ADNI database had LDL/HDL < 2.5.

## Conclusion

We found that the LDL/HDL ratio is associated with AD pathology, cognition, and brain structure and can influence cognitive changes by affecting AD pathology. Although our findings indicate that a higher LDL/HDL ratio is associated with a reduced risk of AD and slower cognitive decline, elevated LDL/HDL levels are generally linked to an increased risk of CVD, which may ultimately heighten the risk of AD. Therefore, improving metabolic and cardiovascular health is essential for effective AD prevention.

## Data Availability

The raw data supporting the conclusions of this article will be made available by the authors, without undue reservation.

## References

[ref1] Abete P. Della-Morte D. Gargiulo G. Basile C. Langellotto A. Galizia G.. (2014). Cognitive impairment and cardiovascular diseases in the elderly. A heart-brain continuum hypothesis. Ageing Res. Rev. 18, 41–52. doi: 10.1016/j.arr.2014.07.003, PMID: 25107566

[ref2] American Diabetes Association (2021). 2. Classification and diagnosis of diabetes: standards of medical Care in Diabetes-2021. Diabetes Care 44, S15–s33. doi: 10.2337/dc21-S002, PMID: 33298413

[ref3] Andrews S. J. Fulton-Howard B. O'Reilly P. Marcora E. Goate A. M. collaborators of the Alzheimer's Disease Genetics Consortium (2021). Causal associations between modifiable risk factors and the Alzheimer's phenome. Ann. Neurol. 89, 54–65. doi: 10.1002/ana.25918, PMID: 32996171 PMC8088901

[ref4] Arsenault B. J. Boekholdt S. M. Kastelein J. J. (2011). Lipid parameters for measuring risk of cardiovascular disease. Nat. Rev. Cardiol. 8, 197–206. doi: 10.1038/nrcardio.2010.223, PMID: 21283149

[ref5] Bailey M. J. Soliman E. Z. McClure L. A. Howard G. Howard V. J. Judd S. E.. (2021). Relation of atrial fibrillation to cognitive decline (from the REasons for geographic and racial differences in stroke [REGARDS] study). Am. J. Cardiol. 148, 60–68. doi: 10.1016/j.amjcard.2021.02.036, PMID: 33684372 PMC9730299

[ref6] Baron R. M. Kenny D. A. (1986). The moderator-mediator variable distinction in social psychological research: conceptual, strategic, and statistical considerations. J. Pers. Soc. Psychol. 51, 1173–1182. doi: 10.1037/0022-3514.51.6.1173, PMID: 3806354

[ref7] de Bruijn R. F. Ikram M. A. (2014). Cardiovascular risk factors and future risk of Alzheimer's disease. BMC Med. 12:130. doi: 10.1186/s12916-014-0130-5, PMID: 25385322 PMC4226863

[ref8] de Galan B. E. Zoungas S. Chalmers J. Anderson C. Dufouil C. Pillai A.. (2009). Cognitive function and risks of cardiovascular disease and hypoglycaemia in patients with type 2 diabetes: the action in diabetes and vascular disease: Preterax and Diamicron modified release controlled evaluation (ADVANCE) trial. Diabetologia 52, 2328–2336. doi: 10.1007/s00125-009-1484-7, PMID: 19688336

[ref9] Expert Panel on Detection, Evaluation, and Treatment of High Blood Cholesterol in Adults (2001). Executive summary of the third report of the National Cholesterol Education Program (NCEP) expert panel on detection, evaluation, and treatment of high blood cholesterol in adults (adult treatment panel III). JAMA 285, 2486–2497. doi: 10.1001/jama.285.19.2486, PMID: 11368702

[ref10] Fernandez M. L. Webb D. (2008). The LDL to HDL cholesterol ratio as a valuable tool to evaluate coronary heart disease risk. J. Am. Coll. Nutr. 27, 1–5. doi: 10.1080/07315724.2008.10719668, PMID: 18460475

[ref11] Fletcher E. Gavett B. Crane P. Soldan A. Hohman T. Farias S.. (2021). A robust brain signature region approach for episodic memory performance in older adults. Brain 144, 1089–1102. doi: 10.1093/brain/awab007, PMID: 33895818 PMC8105039

[ref12] Galea I. (2021). The blood-brain barrier in systemic infection and inflammation. Cell. Mol. Immunol. 18, 2489–2501. doi: 10.1038/s41423-021-00757-x, PMID: 34594000 PMC8481764

[ref13] Jack C. R. Bernstein M. A. Fox N. C. Thompson P. Alexander G. Harvey D.. (2008). The Alzheimer's disease neuroimaging initiative (ADNI): MRI methods. J. Magn. Reson. Imaging 27, 685–691. doi: 10.1002/jmri.21049, PMID: 18302232 PMC2544629

[ref14] Jansson D. Wang M. Thomas R. G. Erickson M. A. Peskind E. R. Li G.. (2022). Markers of cerebrovascular injury, inflammation, and plasma lipids are associated with Alzheimer's disease cerebrospinal fluid biomarkers in cognitively Normal persons. J. Alzheimers Dis. 86, 813–826. doi: 10.3233/JAD-215400, PMID: 35124650 PMC10010435

[ref15] Katsumata Y. Todoriki H. Higashiuesato Y. Yasura S. Ohya Y. Willcox D. C.. (2013). Very old adults with better memory function have higher low-density lipoprotein cholesterol levels and lower triglyceride to high-density lipoprotein cholesterol ratios: KOCOA project. J. Alzheimers Dis. 34, 273–279. doi: 10.3233/JAD-121138, PMID: 23207484 PMC3586553

[ref16] Liu L. Li H. Iyer H. Liu A. J. Zeng Y. Ji J. S. (2021). Apolipoprotein E induced cognitive dysfunction: mediation analysis of lipids and glucose biomarkers in an elderly cohort study. Front. Aging Neurosci. 13:727289. doi: 10.3389/fnagi.2021.727289, PMID: 34483892 PMC8415114

[ref17] McKhann G. Drachman D. Folstein M. Katzman R. Price D. Stadlan E. M. (1984). Clinical diagnosis of Alzheimer's disease: report of the NINCDS-ADRDA work group under the auspices of Department of Health and Human Services Task Force on Alzheimer's disease. Neurology 34, 939–944. doi: 10.1212/WNL.34.7.939, PMID: 6610841

[ref18] Middleton L. T. Riboli E. (2023). Dietary cholesterol and dementia risk. J. Prev Alzheimers Dis. 10, 746–747. doi: 10.14283/jpad.2023.66, PMID: 37874095

[ref19] Muralidharan J. Papandreou C. Soria-Florido M. T. Sala-Vila A. Blanchart G. Estruch R.. (2022). Cross-sectional associations between HDL structure or function, cell membrane fatty acid composition, and inflammation in elderly adults. J. Nutr. 152, 789–795. doi: 10.1093/jn/nxab362, PMID: 34637509

[ref20] Pentikäinen M. O. Oörni K. Ala-Korpela M. Kovanen P. T. (2000). Modified LDL – trigger of atherosclerosis and inflammation in the arterial intima. J. Intern. Med. 247, 359–370. doi: 10.1046/j.1365-2796.2000.00655.x, PMID: 10762453

[ref21] Peters R. Xu Y. Antikainen R. Beckett N. Gussekloo J. Jagger C.. (2021). Evaluation of high cholesterol and risk of dementia and cognitive decline in older adults using individual patient Meta-analysis. Dement. Geriatr. Cogn. Disord. 50, 318–325. doi: 10.1159/000519452, PMID: 34700321

[ref22] Petersen R. C. Aisen P. S. Beckett L. A. Donohue M. C. Gamst A. C. Harvey D. J.. (2010). Alzheimer's disease neuroimaging initiative (ADNI): clinical characterization. Neurology 74, 201–209. doi: 10.1212/WNL.0b013e3181cb3e25, PMID: 20042704 PMC2809036

[ref23] Pfrieger F. W. (2003). Cholesterol homeostasis and function in neurons of the central nervous system. Cell. Mol. Life Sci. 60, 1158–1171. doi: 10.1007/s00018-003-3018-7, PMID: 12861382 PMC11138592

[ref24] Poliakova T. Wellington C. L. (2023). Roles of peripheral lipoproteins and cholesteryl ester transfer protein in the vascular contributions to cognitive impairment and dementia. Mol. Neurodegener. 18:86. doi: 10.1186/s13024-023-00671-y, PMID: 37974180 PMC10652636

[ref25] Racine A. M. Koscik R. L. Nicholas C. R. Clark L. R. Okonkwo O. C. Oh J. M.. (2016). Cerebrospinal fluid ratios with Aβ42 predict preclinical brain β-amyloid accumulation. Alzheimers Demen. 2, 27–38. doi: 10.1016/j.dadm.2015.11.006, PMID: 26955655 PMC4778249

[ref26] Rhea E. M. Banks W. A. (2021). Interactions of lipids, lipoproteins, and apolipoproteins with the blood-brain barrier. Pharm. Res. 38, 1469–1475. doi: 10.1007/s11095-021-03098-6, PMID: 34518942 PMC8901411

[ref27] Saeed A. Lopez O. Cohen A. Reis S. E. (2023). Cardiovascular disease and Alzheimer's disease: the heart-brain Axis. J. Am. Heart Assoc. 12:e030780. doi: 10.1161/JAHA.123.030780, PMID: 37929715 PMC10727398

[ref28] Schnabel R. B. Larson M. G. Yamamoto J. F. Sullivan L. M. Pencina M. J. Meigs J. B.. (2010). Relations of biomarkers of distinct pathophysiological pathways and atrial fibrillation incidence in the community. Circulation 121, 200–207. doi: 10.1161/CIRCULATIONAHA.109.882241, PMID: 20048208 PMC3224826

[ref29] Shaw L. M. Vanderstichele H. Knapik-Czajka M. Clark C. M. Aisen P. S. Petersen R. C.. (2009). Cerebrospinal fluid biomarker signature in Alzheimer's disease neuroimaging initiative subjects. Ann. Neurol. 65, 403–413. doi: 10.1002/ana.21610, PMID: 19296504 PMC2696350

[ref30] Sheng Z. Wang L. Chen M. Zhong F. Wu S. Liang S.. (2025). Cerebrospinal fluid β2-microglobulin promotes the tau pathology through microglia-astrocyte communication in Alzheimer's disease. Alzheimers Res. Ther. 17:2. doi: 10.1186/s13195-024-01665-8, PMID: 39748415 PMC11697900

[ref31] Snider B. J. Fagan A. M. Roe C. Shah A. R. Grant E. A. Xiong C.. (2009). Cerebrospinal fluid biomarkers and rate of cognitive decline in very mild dementia of the Alzheimer type. Arch. Neurol. 66, 638–645. doi: 10.1001/archneurol.2009.55, PMID: 19433664 PMC2759394

[ref32] Song R. Xu H. Dintica C. S. Pan K. Y. Qi X. Buchman A. S.. (2020). Associations between cardiovascular risk, structural brain changes, and cognitive decline. J. Am. Coll. Cardiol. 75, 2525–2534. doi: 10.1016/j.jacc.2020.03.053, PMID: 32439001 PMC10061875

[ref33] Stakos D. A. Stamatelopoulos K. Bampatsias D. Sachse M. Zormpas E. Vlachogiannis N. I.. (2020). The Alzheimer's disease amyloid-Beta hypothesis in cardiovascular aging and disease: JACC focus seminar. J. Am. Coll. Cardiol. 75, 952–967. doi: 10.1016/j.jacc.2019.12.033, PMID: 32130931 PMC7042886

[ref34] Stamatelopoulos K. Sibbing D. Rallidis L. S. Georgiopoulos G. Stakos D. Braun S.. (2015). Amyloid-beta (1-40) and the risk of death from cardiovascular causes in patients with coronary heart disease. J. Am. Coll. Cardiol. 65, 904–916. doi: 10.1016/j.jacc.2014.12.035, PMID: 25744007

[ref35] Turri M. Marchi C. Adorni M. P. Calabresi L. Zimetti F. (1867). Emerging role of HDL in brain cholesterol metabolism and neurodegenerative disorders. Biochim. Biophys. Acta Mol. Cell Biol. Lipids 1867:159123. doi: 10.1016/j.bbalip.2022.159123, PMID: 35151900

[ref36] Wang M. Li Y. Cong L. Hou T. Luo Y. Shi L.. (2021). High-density lipoprotein cholesterol and brain aging amongst rural-dwelling older adults: a population-based magnetic resonance imaging study. Eur. J. Neurol. 28, 2882–2892. doi: 10.1111/ene.14939, PMID: 34031948

[ref37] Ward M. A. Bendlin B. B. McLaren D. G. Hess T. M. Gallagher C. L. Kastman E. K.. (2010). Low HDL cholesterol is associated with lower gray matter volume in cognitively healthy adults. Front. Aging Neurosci. 2:2. doi: 10.3389/fnagi.2010.00029, PMID: 20725527 PMC2914583

[ref38] Wolf H. Hensel A. Arendt T. Kivipelto M. Winblad B. Gertz H. J. (2004). Serum lipids and hippocampal volume: the link to Alzheimer's disease? Ann. Neurol. 56, 745–749. doi: 10.1002/ana.20289, PMID: 15505826

[ref39] Xu W. Tan L. Wang H. F. Jiang T. Tan M. S. Tan L.. (2015). Meta-analysis of modifiable risk factors for Alzheimer's disease. J. Neurol. Neurosurg. Psychiatry 86, jnnp-2015-310548–jnnp-2015-310306. doi: 10.1136/jnnp-2015-310548, PMID: 26294005

[ref40] Yang Z. Lin P. J. Levey A. (2013). Monetary costs of dementia in the United States. N. Engl. J. Med. 369:489. doi: 10.1056/NEJMc1305541, PMID: 23902509

[ref41] Yu Y. Yan P. Cheng G. Liu D. Xu L. Yang M.. (2023). Correlation between serum lipid profiles and cognitive impairment in old age: a cross-sectional study. Gen Psychiatr. 36:e101009. doi: 10.1136/gpsych-2023-101009, PMID: 37144157 PMC10151832

[ref42] Zhang Z. Chen X. Sheng Z. (2024). Association of triglyceride glucose-body mass index with Alzheimer's disease pathology, cognition and brain structure in non-demented people. Sci. Rep. 14:16097. doi: 10.1038/s41598-024-67052-3, PMID: 38997334 PMC11245502

[ref43] Zhang Z. Sheng Z. Liu J. Zhang D. Wang H. Wang L.. (2024). The association of the triglyceride-glucose index with Alzheimer's disease and its potential mechanisms. J. Alzheimers Dis. 102, 77–88. doi: 10.1177/13872877241284216, PMID: 39497312

[ref44] Zhou F. Deng W. Ding D. Zhao Q. Liang X. Wang F.. (2018). High low-density lipoprotein cholesterol inversely relates to dementia in community-dwelling older adults: the Shanghai aging study. Front. Neurol. 9:952. doi: 10.3389/fneur.2018.00952, PMID: 30483213 PMC6240682

[ref45] Zhou Z. Liang Y. Zhang X. Xu J. Lin J. Zhang R.. (2020). Low-density lipoprotein cholesterol and Alzheimer's disease: a systematic review and Meta-analysis. Front. Aging Neurosci. 12:5. doi: 10.3389/fnagi.2020.00005, PMID: 32082137 PMC7002548

[ref46] Zingel R. Bohlken J. Riedel-Heller S. Barth S. Kostev K. (2021). Association between low-density lipoprotein cholesterol levels, statin use, and dementia in patients followed in German general practices. J. Alzheimers Dis. 79, 37–46. doi: 10.3233/JAD-201176, PMID: 33216039

[ref47] Zuccalà G. Cattel C. Manes-Gravina E. Di Niro M. G. Cocchi A. Bernabei R. (1997). Left ventricular dysfunction: a clue to cognitive impairment in older patients with heart failure. J. Neurol. Neurosurg. Psychiatry 63, 509–512. doi: 10.1136/jnnp.63.4.509, PMID: 9343133 PMC2169754

