# Supplementary material for: Association of low-density lipoprotein/high-density lipoprotein ratio with cognition, Alzheimer’s disease biomarkers and brain structure
Source: Front Aging Neurosci. 2025 Apr 30;17:1457160. doi: 10.3389/fnagi.2025.1457160 (PMC12075555; doi:10.3389/fnagi.2025.1457160)
Supplement: Supplementary file 1 [file Data_Sheet_1.docx]

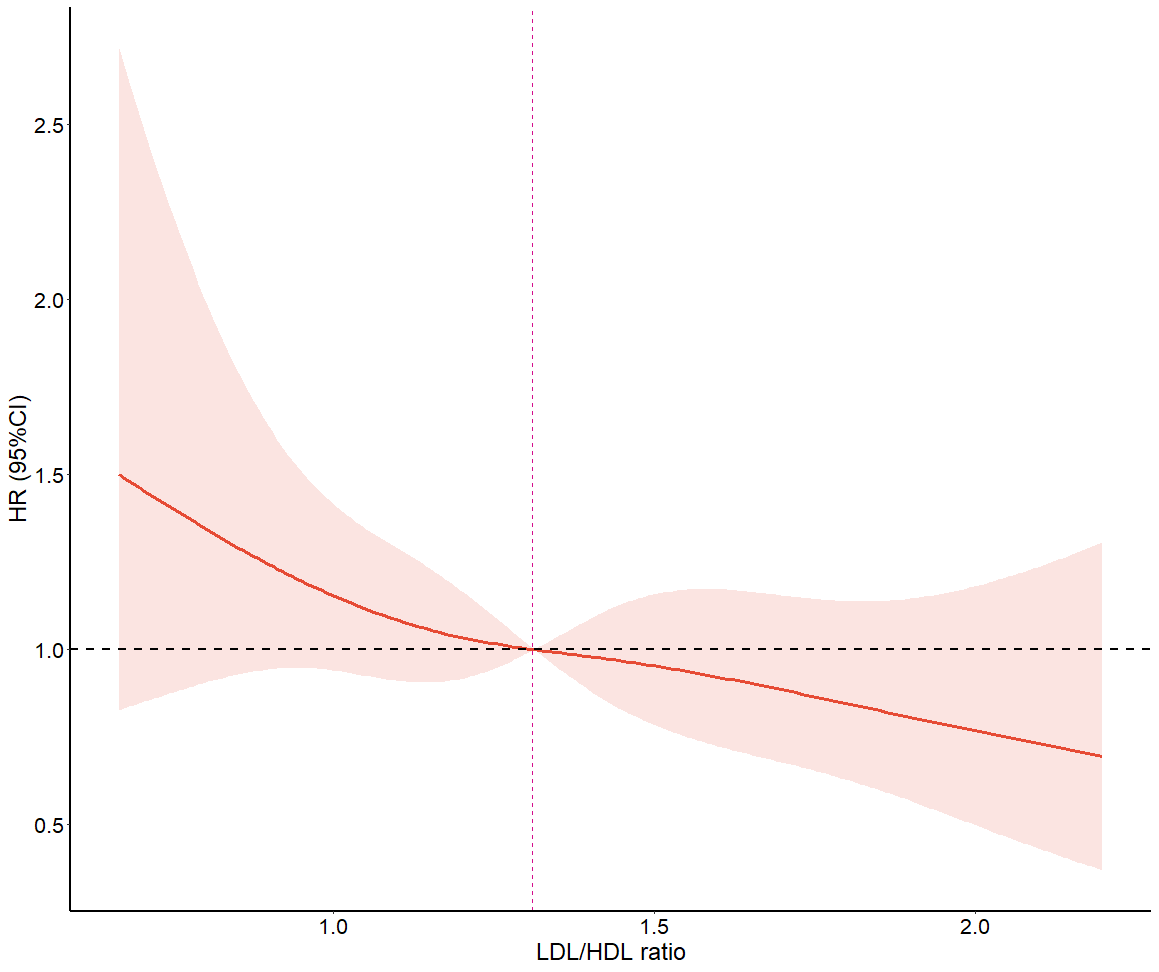
Figure 1 Restricted cubic spline curve for the LDL/HDL ratio and AD hazard ratio

Abbreviations: HR, hazard ratio; CI, confidence interval; LDL/HDL, Low-Density Lipoprotein/High-Density Lipoprotein

Table 1 Collinearity Statistics in ADNI

| **Variables** | **VIF(Aβ42)** | **VIF(Tau)** | **VIF(pTau)** | **VIF(MMSE)** | **VIF(ADAS)** | **VIF(EF)** | **VIF(MEM)** | **VIF(LAN)** |
| --- | --- | --- | --- | --- | --- | --- | --- | --- |
| LDL/HDL | 1.2 | 1.2 | 1.2 | 1.2 | 1.2 | 1.2 | 1.2 | 1.2 |
| Age | 1.1 | 1.1 | 1.1 | 1.1 | 1.1 | 1.1 | 1.1 | 1.1 |
| Sex | 1.3 | 1.3 | 1.3 | 1.3 | 1.3 | 1.3 | 1.3 | 1.3 |
| Education | 1.1 | 1.1 | 1.1 | 1.1 | 1.1 | 1.1 | 1.1 | 1.1 |
| APOEε4 | 1.1 | 1.1 | 1.1 | 1.1 | 1.1 | 1.1 | 1.1 | 1.1 |
| Dignosis | 1.1 | 1.1 | 1.1 | 1.1 | 1.1 | 1.1 | 1.1 | 1.1 |
| Ethnicity | 1.0 | 1.0 | 1.0 | 1.0 | 1.0 | 1.0 | 1.0 | 1.0 |
| Smoking | 1.0 | 1.0 | 1.0 | 1.0 | 1.0 | 1.0 | 1.0 | 1.0 |
| Systolic pressure | 1.3 | 1.3 | 1.3 | 1.3 | 1.3 | 1.3 | 1.3 | 1.3 |
| Diastolic pressure | 1.3 | 1.3 | 1.3 | 1.3 | 1.3 | 1.3 | 1.3 | 1.3 |
| Cardiovascular | 1.1 | 1.1 | 1.1 | 1.1 | 1.1 | 1.1 | 1.1 | 1.1 |
| Diabetes | 1.1 | 1.1 | 1.1 | 1.1 | 1.1 | 1.1 | 1.1 | 1.1 |
| Total Cholesterol | 1.3 | 1.3 | 1.3 | 1.3 | 1.3 | 1.3 | 1.3 | 1.3 |

Abbreviations: ADNI, Alzheimer’s Disease Neuroimaging Initiative; LDL/HDL, Low-Density Lipoprotein/High-Density Lipoprotein; MMSE, mini-mental state examination, ADAS, Alzheimer’s Disease Assessment Scale EF, executive function; MEM, memory function; LAN, language; APOE*ε*4, Apolipoprotein E4.

Table 2 Characteristics based on the LDL/HDL ratio obtained from the Chinese Alzheimer’s Biomaker and Lifestyle (CABLE) study.

|  | Overall |
| --- | --- |
| Number | 1105 |
| Age (years) | 62.96 (10.06) |
| Sex (%) |  |
| Female | 472 (42.7) |
| Male | 633 (57.3) |
| Education (years) | 9.37 (4.40) |
| APOE*ε*4 (%) |  |
| Non-carry | 934 (84.5) |
| Carry | 171 (15.5) |
| Cardiovascular (%) |  |
| No | 615 (55.7) |
| Yes | 490 (44.3) |
| Diabetes (%) |  |
| No | 927 (83.9) |
| Yes | 178 (16.1) |
| Hypertension (%) |  |
| No | 690 (62.4) |
| Yes | 415 (37.6) |
| Smoke (%) |  |
| No | 834 (75.5) |
| Yes | 271 (24.5) |
| Drink (%) |  |
| No | 834 (75.5) |
| Yes | 271 (24.5) |
| Hyperchole- sterolemia |  |
| No | 679 (60.3) |
| Yes | 426 (39.7) |
| Total_C (mmol/L) | 4.87 (1.08) |
| LDL (mmol/L) | 2.90 (0.73) |
| HDL (mmol/L) | 1.20 (0.33) |
| LDL/HDL | 2.50 (0.65) |
| Aβ_40_ (pg/ml) | 6699.84 (3039.46) |
| Aβ_42_ (pg/ml) | 343.33 (220.91) |
| Tau (pg/ml) | 204.45 (101.14) |
| pTau (pg/ml) | 44.73 (15.13) |
| pTau/Aβ_42_ | 0.20 (0.17) |
| Tau/Aβ_42_ | 0.89 (0.86) |
| pTau/Tau | 0.24 (0.08) |
| MMSE score | 27.09 (3.21) |

Abbreviations: LDL/HDL, Low-Density Lipoprotein/High-Density Lipoprotein; APOE*ε*4, Apolipoprotein E4; TOTAL_C, total Cholesterol; Aβ_42_, Amyloid-42; pTau, phosphorylated-tau; Tau, total-tau; APOE4, Apolipoprotein E4; MMSE, mini-mental state examination.

Table 3 Subgroup analysis of association between LDL/HDL ratio and cognition in ADNI.

| Variance | | MMSE | | ADAS | | EF | | MEM | | LAN | | VS | |
| --- | --- | --- | --- | --- | --- | --- | --- | --- | --- | --- | --- | --- | --- |
|  |  | β | P | β | P | β | P | β | P | β | P | β | P |
| Total | | 0.080 | **0.048** | -0.141 | **<0.001** | 0.118 | **0.004** | 0.101 | **0.006** | 0.086 | **0.034** | 0.047 | 0.279 |
| Age | <60 | 0.116 | 0.460 | -0.367 | **0.013** | 0.125 | 0.422 | 0.267 | 0.537 | 0.019 | 0.899 | 0.387 | **0.011** |
|  | ≥60 | 0.058 | 0.171 | -0.102 | **0.015** | 0.098 | **0.024** | 0.058 | 0.132 | 0.071 | 0.099 | 0.011 | 0.805 |
| Sex | Male | 0.049 | 0.350 | -0.093 | 0.088 | 0.102 | 0.629 | 0.058 | 0.247 | 0.014 | 0.788 | 0.011 | 0.852 |
|  | Female | 0.135 | **0.028** | -0.205 | **<0.001** | 0.147 | **0.017** | 0.164 | **0.004** | 0.181 | **0.003** | 0.116 | 0.078 |
| APOEε4 | APOEε4(-) | 0.114 | **0.042** | -0.107 | **0.048** | 0.076 | 0.174 | 0.070 | 0.172 | 0.034 | 0.547 | -0.002 | 0.971 |
|  | APOEε4(+) | 0.024 | 0.697 | -0.163 | **0.009** | 0.135 | **0.032** | 0.122 | **0.033** | 0.139 | **0.024** | 0.088 | 0.178 |
| Diagnosis | CN | -0.015 | 0.851 | -0.144 | 0.061 | 0.064 | 0.406 | 0.094 | 0.202 | -0.009 | 0.906 | -0.016 | 0.844 |
|  | MCI | 0.111 | **0.035** | -0.150 | **0.004** | 0.135 | **0.009** | 0.113 | **0.028** | 0.120 | **0.021** | 0.067 | 0.201 |
| Cardiovascluar | No | 0.106 | 0.154 | -0.150 | **0.034** | 0.158 | **0.028** | 0.139 | **0.037** | 0.160 | **0.027** | 0.096 | 0.211 |
|  | Yes | 0.064 | 0.197 | -0.144 | **0.004** | 0.091 | 0.080 | 0.085 | 0.062 | 0.052 | 0.305 | 0.013 | 0.808 |
| Diabetes | No | 0.071 | 0.108 | -0.135 | **0.002** | 0.094 | **0.035** | 0.093 | **0.018** | 0.090 | **0.041** | 0.045 | 0.338 |
|  | Yes | 0.101 | 0.395 | -0.174 | 0.140 | 0.285 | **0.015** | 0.136 | 0.214 | 0.117 | 0.311 | 0.033 | 0.791 |
| Hyperchole- sterolemia | No | 0.063 | 0.154 | -0.139 | **0.002** | 0.082 | 0.066 | 0.085 | 0.327 | 0.041 | 0.352 | 0.018 | 0.205 |
|  | Yes | 0.295 | **0.024** | -0.084 | 0.520 | 0.163 | 0.269 | 0.111 | 0.395 | 0.198 | 0.155 | 0.091 | 0.510 |

Abbreviations: ADNI, Alzheimer’s Disease Neuroimaging Initiative; LDL/HDL, Low-Density Lipoprotein/High-Density Lipoprotein; MMSE, mini-mental state examination, ADAS, Alzheimer’s Disease Assessment Scale EF, executive function; MEM, memory function; LAN, language; VS, visuospatial functioning; APOE*ε*4, Apolipoprotein E4; CN, cognition normal; MCI, mild cognition mild.

All factors adjusted for age, sex, ethnicity, marriage, education, *APOEε4* carrier status, smoking, cognitive diagnosis, systolic, diastolic, total cholesterol, diabetes and cardiovascular disease except itself.

Table 4 Longitudinal relationship between LDL/HDL ratio with AD pathology, cognitive measures and brain structure.

| Variance | β | P |
| --- | --- | --- |
| Aβ_42_ | 0.001 | 0.844 |
| Tau | -0.002 | 0.698 |
| pTau | -0.002 | 0.685 |
| Tau/Aβ_42_ | -0.007 | 0.427 |
| pTau/Aβ_42_ | -0.006 | 0.438 |
| pTau/Tau | 0.003 | 0.799 |
| MMSE | 0.026 | **0.047** |
| ADAS | -0.023 | 0.054 |
| MEM | 0.015 | **0.033** |
| EF | 0.005 | 0.467 |
| LAN | 0.017 | **0.048** |
| VS | -0.009 | 0.330 |
| Ventricles | -0.002 | 0.650 |
| Hippocampus | -0001 | 0.724 |
| WholeBrain | -0.004 | 0.161 |
| Fusiform | 0.004 | 0.452 |
| Entorhinal | -0.009 | 0.177 |
| Middle temporal | -0.002 | 0.774 |

Abbreviations: LDL/HDL, Low-Density Lipoprotein/High-Density Lipoprotein; AD, Alzheimer’s Disease; Aβ_42_, Amyloid-42; pTau, phosphorylated-tau; Tau, total-tau; MMSE, mini-mental state examination, ADAS, Alzheimer’s Disease Assessment Scale; EF, executive function; MEM, memory function; LAN, language; VS, visuospatial functioning.

All factors adjusted for age, sex, ethnicity, marriage, education, *APOEε4* carrier status, smoking, cognitive diagnosis, systolic, diastolic, total cholesterol, diabetes and cardiovascular disease.

Table 5 Subgroup analysis of association between LDL/HDL ratio and AD biomarkers in ADNI.

| Variance | | Aβ42 | | Tau | | pTau | | pTau/Aβ42 | | Tau/Aβ42 | | pTau/Tau | |
| --- | --- | --- | --- | --- | --- | --- | --- | --- | --- | --- | --- | --- | --- |
|  |  | β | P | β | P | β | P | β | P | β | P | β | P |
| Total | | 0.108 | **0.009** | -0.140 | **<0.001** | -0.141 | **<0.001** | -0.145 | **<0.001** | -0.147 | **<0.001** | -0.101 | **0.018** |
| Age | <60 | 0.120 | 0.390 | -0.111 | 0.412 | -0.121 | 0.385 | -0.190 | 0.183 | -0.186 | 0.185 | -0.126 | 0.431 |
|  | ≥60 | 0.088 | **0.045** | -0.141 | **0.001** | -0.138 | **0.001** | -0.128 | **0.002** | -0.131 | **0.002** | -0.084 | 0.059 |
| Sex | Male | 0.067 | 0.209 | -0.151 | **0.005** | -0.161 | **0.003** | -0.163 | **0.002** | -0.155 | **0.003** | -0.135 | **0.014** |
|  | Female | 0.180 | **0.004** | -0.121 | **0.048** | -0.110 | 0.072 | -0.122 | **0.044** | -0.138 | **0.021** | -0.023 | 0.418 |
| APOEε4 | APOEε4(-) | 0.056 | 0.361 | -0.152 | **0.012** | -0.154 | **0.011** | -0.137 | **0.024** | -0.136 | **0.025** | -0.096 | 0.120 |
|  | APOEε4(+) | 0.174 | **0.008** | -0.130 | **0.042** | -0.130 | **0.042** | -0.153 | **0.017** | -0.160 | **0.012** | -0.098 | 0.138 |
| Diagnosis | CN | 0.010 | 0.901 | -0.177 | **0.022** | -0.166 | **0.034** | -0.141 | 0.074 | -0.145 | 0.066 | -0.016 | 0.838 |
|  | MCI | 0.154 | **0.002** | -0.137 | **0.006** | -0.143 | **0.004** | -0.152 | **0.002** | -0.152 | **0.002** | -0.131 | **0.011** |
| Cardiovascluar | No | 0.054 | 0.455 | -0.169 | **0.023** | -0.158 | **0.034** | -0.123 | 0.086 | -0.135 | 0.058 | -0.066 | 0.386 |
|  | Yes | 0.125 | **0.015** | -0.138 | **0.006** | -0.144 | **0.004** | -0.156 | **0.002** | -0.152 | **0.002** | -0.121 | **0.022** |
| Diabetes | No | 0.114 | **0.010** | -0.126 | **0.004** | -0.128 | **0.004** | -0.132 | **0.002** | -0.133 | **0.002** | -0.081 | 0.076 |
|  | Yes | 0.105 | 0.344 | -0.233 | **0.042** | -0.226 | **0.050** | -0.234 | **0.032** | -0.245 | **0.023** | -0.205 | 0.075 |
| Hyperchole- sterolemia | No | 0.110 | **0.015** | -0.165 | **<0.001** | -0.165 | **<0.001** | -0.160 | **<0.001** | -0.161 | **<0.001** | -0.114 | **0.014** |
|  | Yes | 0.064 | 0.642 | -0.003 | 0.978 | 0.028 | 0.81 | 0.009 | 0.941 | -0.008 | 0.949 | 0.092 | 0.490 |

Abbreviations: ADNI, Alzheimer’s Disease Neuroimaging Initiative; LDL/HDL, Low-Density Lipoprotein/High-Density Lipoprotein; Aβ_42_, Amyloid-42; pTau, phosphorylated-tau; Tau, total-tau; APOE*ε*4, Apolipoprotein E4; CN, cognition normal; MCI, mild cognition mild.

All factors adjusted for age, sex, ethnicity, marriage, education, *APOEε4* carrier status, smoking, cognitive diagnosis, systolic, diastolic, total cholesterol, diabetes and cardiovascular disease except itself.

Table 6 Subgroup analysis of association between LDL/HDL ratio and AD biomarkers as well as cognition in CABLE.

| Variance | Aβ40 | | Aβ42 | | Tau | | pTau | | pTau/Aβ42 | | Tau/Aβ42 | | pTau/Tau | | MMSE | |
| --- | --- | --- | --- | --- | --- | --- | --- | --- | --- | --- | --- | --- | --- | --- | --- | --- |
|  | β | P | β | P | β | P | β | P | β | P | β | P | β | P | β | P |
| Total | 0.028 | 0.406 | 0.029 | 0.37 | -0.064 | **0.046** | -0.085 | **0.008** | -0.034 | 0.291 | -0.01 | 0.344 | 0.019 | 0.557 | 0.007 | 0.804 |
| Age |  |  |  |  |  |  |  |  |  |  |  |  |  |  |  |  |
| <60 | 0.030 | 0.610 | 0.035 | 0.548 | -0.046 | 0.439 | -0.033 | 0.583 | 0.021 | 0.717 | 0.010 | 0.869 | 0.049 | 0.412 | -0.012 | 0.827 |
| ≥60 | 0.025 | 0.542 | 0.026 | 0.500 | -0.075 | 0.063 | -0.112 | **0.005** | -0.051 | 0.208 | -0.041 | 0.308 | -0.018 | 0.655 | 0.015 | 0.686 |
| Sex |  |  |  |  |  |  |  |  |  |  |  |  |  |  |  |  |
| Male | 0.061 | 0.156 | 0.024 | 0.557 | -0.076 | 0.070 | -0.091 | 0.029 | -0.031 | 0.459 | -0.023 | 0.583 | 0.009 | 0.834 | -0.018 | 0.647 |
| Female | -0.026 | 0.623 | 0.034 | 0.513 | -0.057 | 0.263 | -0.076 | 0.143 | -0.031 | 0.559 | -0.040 | 0.448 | 0.051 | 0.327 | 0.046 | 0.321 |
| APOE*ε*4 |  |  |  |  |  |  |  |  |  |  |  |  |  |  |  |  |
| APOE*ε*4(-) | 0.055 | 0.124 | 0.028 | 0.412 | -0.047 | 0.176 | -0.062 | 0.078 | -0.013 | 0.716 | -0.009 | 0..800 | 0.020 | 0.583 | -0.007 | 0.812 |
| APOE*ε*4(+) | -0.121 | 0.166 | 0.015 | 0.861 | -0.166 | **0.046** | -0.243 | **0.004** | -0.142 | 0.099 | -0.150 | 0.081 | 0.024 | 0.777 | 0.082 | 0.308 |
| Cardiovascluar |  |  |  |  |  |  |  |  |  |  |  |  |  |  |  |  |
| No | 0.015 | 0.735 | 0.018 | 0.668 | -0.042 | 0.320 | -0.051 | 0.235 | -0.028 | 0.516 | -0.028 | 0.519 | 0.025 | 0.567 | 0.046 | 0.240 |
| Yes | 0.053 | 0.300 | 0.047 | 0.334 | -0.089 | 0.074 | -0.128 | **0.011** | -0.048 | 0.344 | -0.042 | 0.406 | 0.022 | 0.662 | -0.029 | 0.506 |
| Diabetes |  |  |  |  |  |  |  |  |  |  |  |  |  |  |  |  |
| No | 0.036 | 0.310 | 0.018 | 0.591 | -0.086 | **0.013** | -0.087 | **0.012** | -0.022 | 0.533 | -0.022 | 0.534 | 0.036 | 0.313 | 0.021 | 0.499 |
| Yes | -0.017 | 0.843 | 0.058 | 0.502 | 0.051 | 0.558 | -0.053 | 0.546 | -0.069 | 0.428 | -0.044 | 0.608 | -0.151 | 0.076 | -0.119 | 0.124 |
| Hyperchole-sterolemia |  |  |  |  |  |  |  |  |  |  |  |  |  |  |  |  |
| No | -0.011 | 0.825 | 0.069 | 0.143 | -0.130 | **0.005** | -0.133 | **0.004** | -0.066 | 0.168 | -0.075 | 0.116 | 0.088 | 0.067 | 0.053 | 0.218 |
| Yes | 0.110 | **0.031** | 0.035 | 0.471 | -0.006 | 0.900 | -0.034 | 0.503 | 0.001 | 0.832 | 0.029 | 0.571 | -0.065 | 0.198 | 0.022 | 0.615 |

Abbreviations: CABLE, the Chinese Alzheimer’s Biomaker and Lifestyle study; LDL/HDL, Low-Density Lipoprotein/High-Density Lipoprotein; APOE*ε*4, Apolipoprotein E4; Aβ_42_, Amyloid-42; pTau, phosphorylated-tau; Tau, total-tau; MMSE, mini-mental state examination; APOE*ε*4, Apolipoprotein E4; CN, cognition normal; MCI, mild cognition mild.

All factors adjusted for age, sex, education, *APOEε4* carrier status, smoking, hypertension, total cholesterol, diabetes and cardiovascular disease except itself.

Table 7 Subgroup analysis of association between LDL/HDL ratio and brain structure in ADNI.

| Variance | | Ventricles | | Hippocampus | | WholeBrain | | Entorhinal cortex | | Fusiform gyrus | | Middle temporal lobe | |
| --- | --- | --- | --- | --- | --- | --- | --- | --- | --- | --- | --- | --- | --- |
|  |  | β | P | β | P | β | P | β | P | β | P | β | P |
| Total | | -0.038 | 0.362 | 0.102 | **0.027** | 0.057 | 0.155 | 0.093 | **0.041** | 0.030 | 0.513 | 0.104 | **0.021** |
| Age | <60 | -0.712 | 0.308 | -0.074 | 0.950 | -0.353 | 0.569 | 0.082 | 0.906 | -0.034 | 0.951 | 0.019 | 0.977 |
|  | ≥60 | -0.039 | 0.367 | 0.098 | **0.035** | 0.055 | 0.179 | 0.091 | **0.049** | 0.025 | 0.585 | 0.100 | **0.027** |
| Sex | Male | -0.039 | 0.515 | 0.089 | 0.152 | 0.075 | 0.197 | 0.070 | 0.258 | 0.033 | 0.608 | 0.065 | 0.305 |
|  | Female | -0.043 | 0.468 | 0.163 | **0.007** | 0.047 | 0.477 | 0.132 | 0.056 | 0.035 | 0.614 | 0.162 | **0.023** |
| APOE*ε*4 | APOE*ε*4(-) | -0.034 | 0.574 | 0.055 | 0.409 | 0.041 | 0.470 | 0.072 | 0.255 | -0.020 | 0.748 | 0.068 | 0.269 |
|  | APOE*ε*4(+) | -0.041 | 0.508 | 0.092 | 0.281 | 0.050 | 0.372 | 0.092 | 0.181 | 0.042 | 0.514 | 0.105 | 0.102 |
| Diagnosis | CN | -0.096 | 0.244 | 0.125 | **0.023** | -0.080 | 0.292 | 0.141 | 0.096 | -0.105 | 0.196 | -0.039 | 0.618 |
|  | MCI | -0.012 | 0.817 | 0.109 | 0.057 | 0.094 | **0.046** | 0.070 | 0.207 | 0.062 | 0.258 | 0.140 | **0.010** |
| Cardiovascluar | No | -0.035 | 0.637 | 0.209 | **0.009** | 0.098 | 0.171 | 0.146 | 0.061 | 0.121 | 0.122 | 0.198 | **0.010** |
|  | Yes | -0.033 | 0.531 | 0.051 | 0.369 | 0.034 | 0.489 | 0.083 | 0.145 | -0.019 | 0.744 | 0.053 | 0.358 |
| Diabetes | No | -0.039 | 0.393 | 0.077 | 0.119 | 0.022 | 0.613 | 0.074 | 0.131 | 0.007 | 0.893 | 0.089 | 0.068 |
|  | Yes | -0.081 | 0.490 | 0.254 | **0.031** | 0.263 | **0.014** | 0.191 | 0.115 | 0.180 | 0.125 | 0.186 | 0.132 |
| Hyperchole- sterolemia | No | -0.011 | 0.830 | 0.079 | 0.117 | **0.015** | 0.740 | 0.069 | 0.175 | 0.010 | 0.841 | 0.063 | 0.208 |
|  | Yes | -0.130 | 0.367 | 0.044 | 0.778 | 0.093 | 0.481 | 0.041 | 0.772 | -0.117 | 0.391 | 0.056 | 0.674 |

Abbreviations: ADNI, Alzheimer’s Disease Neuroimaging Initiative; LDL/HDL, Low-Density Lipoprotein/High-Density Lipoprotein; APOE*ε*4, Apolipoprotein E4; CN, cognition normal; MCI, mild cognition mild.

All factors adjusted for age, sex, ethnicity, marriage, education, *APOEε4* carrier status, smoking, cognitive diagnosis, systolic, diastolic, total cholesterol, diabetes and cardiovascular disease except itself.

Table 8 Mediation analyses of LDL/HDL and cognitive measurements with AD biomarkers as mediators in non-dementia participants.

| Mediation |  | a | P | b | P | c | P | c' | P | Proportion(%) | P |
| --- | --- | --- | --- | --- | --- | --- | --- | --- | --- | --- | --- |
| Aβ_42_ | MMSE | 10.6 | 0.009 | 0.001 | <0.001 | 0.3894 | 0.047 | 0.275 | 0.148 | 26.68% | 0.066 |
|  | ADAS | 10.6 | 0.009 | -0.003 | <0.001 | -1.802 | <0.001 | -1.478 | 0.003 | 17.54% | **0.008** |
|  | EF | 10.6 | 0.009 | 6.75E-4 | <0.001 | 0.285 | 0.004 | 0.215 | 0.026 | 24.00% | **0.014** |
|  | MEM | 10.6 | 0.009 | 5.61E-4 | <0.001 | 0.21 | 0.006 | 0.153 | 0.039 | 27.19% | **0.008** |
|  | LAN | 10.6 | 0.009 | 4.74E-4 | <0.001 | 0.185 | 0.034 | 0.137 | 0.112 | 25.38% | **0.038** |
|  | VS | 10.6 | 0.009 | 2.43E-4 | 0.003 | 0.087 | 0.279 | 0.062 | 0.44 | 20.39% | 0.280 |
| Tau | MMSE | -47.68 | <0.001 | -0.003 | <0.001 | 0.384 | 0.047 | 0.261 | 0.174 | 31.37% | 0.056 |
|  | ADAS | -47.68 | <0.001 | 0.007 | <0.001 | -1.802 | <0.001 | -1.475 | 0.004 | 18.00% | **0.002** |
|  | EF | -47.68 | <0.001 | -0.002 | <0.001 | 0.285 | 0.004 | 0.212 | 0.031 | 25.18% | **0.004** |
|  | MEM | -47.68 | <0.001 | -0.002 | <0.001 | 0.21 | 0.006 | 0.135 | 0.07 | 35.50% | **0.004** |
|  | LAN | -47.68 | <0.001 | -0.001 | <0.001 | 0.185 | 0.034 | 0.131 | 0.131 | 27.74% | **0.032** |
|  | VS | -47.68 | <0.001 | -5.7E-4 | 0.015 | 0.087 | 0.279 | 0.061 | 0.450 | 21.54% | 0.242 |
| pTau | MMSE | -5.370 | <0.001 | -0.024 | <0.001 | 0.384 | 0.047 | 0.258 | 0.179 | 31.65% | 0.060 |
|  | ADAS | -5.370 | <0.001 | 0.070 | <0.001 | -1.802 | <0.001 | -1.457 | 0.004 | 18.68% | **<0.001** |
|  | EF | -5.370 | <0.001 | -0.014 | <0.001 | 0.285 | 0.004 | 0.211 | 0.032 | 24.60% | **0.002** |
|  | MEM | -5.370 | <0.001 | -0.015 | <0.001 | 0.210 | 0.006 | 0.133 | 0.073 | 36.45% | **0.006** |
|  | LAN | -5.370 | <0.001 | -0.010 | <0.001 | 0.185 | 0.034 | 0.133 | 0.127 | 27.32% | **0.034** |
|  | VS | -5.370 | <0.001 | -0.005 | 0.012 | 0.087 | 0.279 | 0.060 | 0.457 | 24.41% | 0.282 |
| pTau/Aβ_42_ | MMSE | -0.011 | <0.001 | -0.144 | <0.001 | 0.384 | 0.047 | 0.227 | 0.235 | 39.45% | **0.046** |
|  | ADAS | -0.011 | <0.001 | 43.136 | <0.001 | -1.802 | <0.001 | -1.346 | 0.007 | 25.40% | **0.002** |
|  | EF | -0.011 | <0.001 | -8.772 | <0.001 | 0.285 | 0.004 | 0.190 | 0.051 | 32.99% | **0.003** |
|  | MEM | -0.011 | <0.001 | -4.832 | <0.001 | 0.21 | 0.006 | 0.118 | 0.107 | 43.92% | **0.007** |
|  | LAN | -0.011 | <0.001 | -6.428 | <0.001 | 0.185 | 0.034 | 0.115 | 0.180 | 36.66% | **0.048** |
|  | VS | -0.011 | <0.001 | -3.132 | 0.003 | 0.087 | 0.279 | 0.053 | 0.511 | 28.50% | 0.316 |
| Tau/Aβ_42_ | MMSE | -0.105 | <0.001 | -1.633 | <0.001 | 0.384 | 0.047 | 0.218 | 0.253 | 41.78% | **0.041** |
|  | ADAS | -0.105 | <0.001 | 4.908 | <0.001 | -1.802 | <0.001 | -1.318 | 0.009 | 26.80% | **<0.001** |
|  | EF | -0.105 | <0.001 | -1.001 | <0.001 | 0.384 | 0.004 | 0.184 | 0.058 | 34.19% | **0.006** |
|  | MEM | -0.105 | <0.001 | -0.955 | <0.001 | 0.210 | 0.006 | 0.113 | 0.121 | 46.09% | **0.007** |
|  | LAN | -0.105 | <0.001 | -0.757 | <0.001 | 0.185 | 0.034 | 0.109 | 0.206 | 40.16% | **0.032** |
|  | VS | -0.105 | <0.001 | -0.352 | 0.002 | 0.087 | 0.279 | 0.052 | 0.524 | 28.65% | 0.285 |
| pTau/Tau | MMSE | -0.003 | 0.018 | -31.226 | <0.001 | 0.384 | 0.047 | 0.308 | 0.108 | 19.03% | 0.058 |
|  | ADAS | -0.003 | 0.018 | 89.395 | <0.001 | -1.802 | <0.001 | -1.592 | 0.002 | 11.54% | **0.022** |
|  | EF | -0.003 | 0.018 | -16.235 | <0.001 | 0.285 | 0.004 | 0.246 | 0.013 | 13.25% | **0.020** |
|  | MEM | -0.003 | 0.018 | -16.812 | <0.001 | 0.210 | 0.006 | 0.169 | 0.024 | 19.05% | **0.019** |
|  | LAN | -0.003 | 0.018 | -8.834 | 0.008 | 0.185 | 0.034 | 0.165 | 0.060 | 10.19% | **0.050** |
|  | VS | -0.003 | 0.018 | -7.616 | 0.014 | 0.087 | 0.279 | 0.069 | 0.394 | 14.40% | 0.286 |
|  |  |  |  |  |  |  |  |  |  |  |  |

Abbreviations: LDL/HDL, Low-Density Lipoprotein/High-Density Lipoprotein; AD, Alzheimer’s Disease; Aβ_42_, Amyloid-42; pTau, phosphorylated-tau; Tau, total-tau; MMSE, mini-mental state examination, ADAS11, Alzheimer’s Disease Assessment Scale 11; ADAS13, Alzheimer’s Disease Assessment Scale 13; EF, executive function; MEM, memory function; LAN, language; VS, visuospatial functioning.

Additional notes on the Chine Alzheimer’s Biomaker and Lifestyle (CABLE) study.

Since 2017, CABLE has been a large-scale study aiming to explore AD’s risk factors and biomarkers in the Han Chinese population. Participants were recruited from Qingdao Municipal Hospital, Shandong, China. Inclusion criteria comprised 1) self-reported Han Chinese in origin and 2) age between 40 and 90 years. The exclusion criteria were 1) major neurological disorders, such as infection, trauma, epilepsy, or multiple sclerosis; 2) major psychological disorders, such as major depressive disorder or general anxiety disorder; 3) malignant tumors; and 4) genetic disorders. All participants underwent neuropsychological examinations and biological sample (blood and CSF sample) collection by doctors with standardized training via a structured questionnaire and an electronic medical record system.
